# Laser-Assisted *In Vitro* Fertilization Facilitates Fertilization of Vitrified-Warmed C57BL/6 Mouse Oocytes with Fresh and Frozen-Thawed Spermatozoa, Producing Live Pups

**DOI:** 10.1371/journal.pone.0091892

**Published:** 2014-03-11

**Authors:** Stephanie E. Woods, Peimin Qi, Elizabeth Rosalia, Tony Chavarria, Allan Discua, John Mkandawire, James G. Fox, Alexis García

**Affiliations:** Transgenic Core Facility, Division of Comparative Medicine, Massachusetts Institute of Technology, Cambridge, Massachusetts, United States of America; University Hospital of Münster, Germany

## Abstract

The utility of cryopreserved mouse gametes for reproduction of transgenic mice depends on development of assisted reproductive technologies, including vitrification of unfertilized mouse oocytes. Due to hardening of the zona pellucida, spermatozoa are often unable to penetrate vitrified-warmed (V-W) oocytes. Laser-assisted *in vitro* fertilization (LAIVF) facilitates fertilization by allowing easier penetration of spermatozoa through a perforation in the zona. We investigated the efficiency of V-W C57BL/6NTac oocytes drilled by the XYClone laser, compared to fresh oocytes. By using DAP213 for cryoprotection, 83% (1,470/1,762) of vitrified oocytes were recovered after warming and 78% were viable. Four groups were evaluated for two-cell embryo and live offspring efficiency: 1) LAIVF using V-W oocytes, 2) LAIVF using fresh oocytes, 3) conventional IVF using V-W oocytes and 4) conventional IVF using fresh oocytes. First, the groups were tested using fresh C57BL/6NTac spermatozoa (74% motile, 15 million/ml). LAIVF markedly improved the two-cell embryo efficiency using both V-W (76%, 229/298) and fresh oocytes (69%, 135/197), compared to conventional IVF (7%, 12/182; 6%, 14/235, respectively). Then, frozen-thawed C57BL/6NTac spermatozoa (35% motile, 15 million/ml) were used and LAIVF was again found to enhance fertilization efficiency, with two-cell embryo rates of 87% (298/343) using V-W oocytes (P<0.05, compared to fresh spermatozoa), and 73% (195/266) using fresh oocytes. Conventional IVF with frozen-thawed spermatozoa using V-W (6%, 10/168) and fresh (5%, 15/323) oocytes produced few two-cell embryos. Although live offspring efficiency following embryo transfer was greater with conventional IVF (35%, 18/51; LAIVF: 6%, 50/784), advantage was seen with LAIVF in live offspring obtained from total oocytes (5%, 50/1,010; conventional IVF: 2%, 18/908). Our results demonstrated that zona-drilled V-W mouse oocytes can be used for IVF procedures using both fresh and frozen-thawed spermatozoa, producing live pups. The ability to cryopreserve mouse gametes for LAIVF may facilitate management of large-scale transgenic mouse production facilities.

## Introduction

Transgenic mice are used broadly in biomedical research and the utility of cryopreserved mouse gametes for reproduction depends on development of assisted reproductive technologies, including vitrification of unfertilized mouse oocytes. In contrast to another cryopreservation technique called ‘slow-freezing’, vitrification prevents physiological damage caused by the formation of ice crystals both within and outside the cells, and reduces chilling damage by allowing for more rapid freezing [1], [2]. This technique is established for cryopreservation of mammalian embryos, but is a developing technology for oocytes [1], [3], [4]. The recent development of a simple, efficient and general-purpose protocol for vitrification of mouse oocytes using DAP213 (2 M DMSO, 1 M acetamide, 3 M propylene glycol) has made possible further applications of vitrified-warmed (V-W) oocytes in both large- and small- scale transgenic mouse production facilities [5], [6].

Spermatozoa are often unable to penetrate V-W mouse oocytes due to hardening of the zona pellucida (ZP) following premature release of cortical granules [7]–[10]. Previous studies have evaluated intracytoplasmic sperm injection (ICSI) [9], partial ZP digestion [7] and piezo-actuated zona drilling [8] or incision [10] for easier penetration, and found fertilization advantage. One study also examined laser-assisted zona drilling prior to vitrification of mouse oocytes and demonstrated improved post-warming *in vitro* fertilization (IVF) using spermatozoa from a subfertile transgenic mouse [11].

Laser-assisted zona drilling offers promise for assisted reproduction in both humans and mice [11]–[17]. A recent study using laser-assisted IVF (LAIVF) to derive subfertile genetically-modified (GM) mouse lines achieved fertilization rates 4 to 10 times that of conventional IVF [12]. Further, fertilization rates have been shown to be higher with LAIVF in mice with poor quality sperm [11], [13], [16]–[19].

To test whether zona-drilled V-W mouse oocytes can be used for IVF procedures using both fresh and frozen-thawed spermatozoa, we investigated the fertilization efficiency (% two-cell embryos) and % live offspring of V-W C57BL/6NTac oocytes zona-drilled by the XYClone laser, compared to fresh oocytes. Our study suggests that laser-assisted zona drilling after vitrification of mouse oocytes for IVF may facilitate the production of transgenic mice.

## Methods

### Ethics Statement

Mice were housed in an Association for Assessment and Accreditation of Laboratory Animal Care International-accredited facility. This study was carried out in strict accordance with the recommendations in the Guide for the Care and Use of Laboratory Animals. All animal use was approved by the M.I.T. Committee on Animal Care (Animal Welfare Assurance #: A-3125-01).

### Mice

Four-month-old C57BL/6NTac proven breeder males were used for collection of spermatozoa, and 4–5-week-old C57BL/6NTac superovulated females were used for oocyte collection. Two-cell embryo transfer recipients were 0.5 dpc (days post coitum) pseudopregnant Crl:CD1(ICR) females (2–4 months old). Vasectomized male mice [Crl:CD1(ICR); 2–8 months old] were used to induce pseudopregnancies. Macroenvironmental housing conditions included a 14∶10 and 12∶12 light/dark cycle for oocyte donors and two-cell embryo recipients, respectively, with temperature maintenance at 68±2**°**F.

### Media

All media used for spermatozoa collection and evaluation, oocyte collection and warming, and conventional and laser-assisted IVF were equilibrated and warmed prior to use. An incubator (Forma Scientific, Inc., Marietta, OH) set at 37**°**C with 5% CO_2_ was used throughout the experiment for pre-warming and incubation. Potassium simplex optimized medium (KSOMaa) and human tubal fluid medium (HTF) were purchased from Zenith Biotech (Guilford, CT); bovine serum albumin (Sigma-Aldrich, Saint Louis, MO) was added to the media at 4 mg/ml. FHM medium, a modified KSOM medium using HEPES instead of bicarbonate as the buffer, and M2 medium were both purchased from Millipore (Billerica, MA).

### Spermatozoa Collection, Cryopreservation and Analysis

For collection of fresh spermatozoa after CO_2_ euthanasia of the donor, the epididymides and vasa deferentia were removed and placed into a 200 µl drop of HTF medium. Multiple tears in the epididymides and vasa deferentia were made using an 18-gauge needle under light microscopy, and spermatozoa were left to swim out into the medium during a 10-minute incubation period at 37**°**C. Spermatozoa collected for cryopreservation was obtained similarly from a donor, but into a 200 µl drop of cryoprotectant agent (CPA; 3% milk, 18% raffinose, 488 mM L-glutamine). Ten µl of spermatozoa suspension was loaded into each 0.25 ml plastic straw. Straws were slowly cooled in liquid nitrogen vapor (about −150**°**C) for 10–15 minutes, then plunged into liquid nitrogen for storage; spermatozoa were kept frozen for at least 1 week prior to use.

One person was responsible for evaluating concentration and motility of both fresh and frozen-thawed (pre- and post-freeze) spermatozoa. Spermatozoa concentration of suspension was determined by adding 4 µl of the spermatozoa in HTF medium or CPA into 96 µl of sterile water (dilution factor of 25). Ten µl of homogenized, diluted spermatozoa were loaded under the coverslip on each side of an improved Neubauer hemocytometer (Bright-Line, American Optical, Buffalo, NY.) and placed into a pre-wetted incubation chamber for 20 minutes prior to analysis. Spermatozoa were counted in the 5 designated squares under light microscopy at 100x magnification and the count averaged between the two sides, with concentration per ml calculated as dilution factor *count in 5 squares *0.05×10^6^ [20].

To determine motility of spermatozoa, 4 µl of the spermatozoa suspended in HTF medium or CPA was added to 96 µl of HTF medium and incubated at 37**°**C with 5% CO_2_ for 10 minutes. Following incubation, 10 µl of spermatozoa uniformly dispersed in HTF medium was pipetted onto a microscope slide and covered with a coverslip. At 100x magnification, the first 100 spermatozoa manually visualized were counted to establish % progressively motile, % non-progressively motile and % immotile. Based on visual observation, progressive motility was defined as spermatozoa swimming in a forward manner [21].

### Oocyte Collection

Pregnant mare serum gonadotropin (National Hormone and Peptide Program, Torrance, CA) and human chorionic gonadotropin (hCG; Sigma-Aldrich, Saint Louis, MO) were administered intraperitoneally (5 IU per mouse) 48 hours apart to superovulate oocyte donors. Oviducts were collected from females euthanized by cervical dislocation starting at 13 hours after hCG administration; approximately 20–30 oocytes are collected per donor. Cumulus oocyte complexes (COCs) were released from the oviduct ampullas into HTF (for conventional IVF) or FHM (for LAIVF) medium containing 0.1% hyaluronidase (HA). The dish of COCs with medium containing HA was incubated for 5 minutes at 37**°**C to remove cumulus cells from oocytes. Cumulus-free oocytes were then collected and washed 3 times with HTF or FHM medium.

### Vitrification and Warming of Oocytes

For oocyte vitrification, a modified Nakagata et al. protocol was followed [5], [6]. Cumulus-free oocytes were equilibrated in a drop of FHM medium containing 1 mM DMSO (DMSO/FHM) at room temperature, and loaded into a cryogenic vial to then sit at 4**°**C for 3–5 minutes; each vial contained approximately 50 oocytes in 5 µl DMSO/FHM. Ninety microliters of 4**°**C cryoprotectant DAP213 (2 M DMSO, 1 M acetamide, 3 M propylene glycol) was added to the vial and the lid was loosely closed. The vial was then immediately submerged in liquid nitrogen and stored until use, for at least 1 week. The vitrification procedure took less than 10 minutes.

On the morning of IVF, vials were periodically removed from liquid nitrogen; within 30 seconds, 0.9 ml of pre-warmed (37**°**C) FHM medium containing 0.25 M sucrose (sucrose/FHM) was added and mixed by gently pipetting up-and-down. An additional 0.5 ml of sucrose/FHM medium was used to rinse each vial to maximize oocyte recovery. Medium containing oocytes was placed in a 35 mm dish for evaluation. Survived V-W oocytes were washed 2 times with pre-equilibrated HTF or FHM medium and used for conventional IVF or LAIVF, as described below.

### Conventional and Laser-assisted IVF

On the morning of IVF, the straws of frozen spermatozoa were thawed in a 37**°**C water bath for 2 minutes. The ends of the straws were cut and the contents of one straw (10 µl; approximately 3 million spermatozoa) were purged into a 200 µl IVF drop of HTF medium (15 million spermatozoa/ml). For IVF using fresh spermatozoa, 10 µl of gently mixed spermatozoa suspension (approximately 3 million spermatozoa) was added to each 200 µl IVF drop of HTF medium (15 million spermatozoa/ml). Frozen-thawed and fresh spermatozoa were allowed to pre-incubate in the IVF drop for at least 20 minutes prior to the addition of oocytes.

For conventional IVF, approximately 100 survived V-W oocytes or freshly harvested cumulus-free oocytes were incubated for 4–6 hours in a 200 µl IVF drop of HTF medium containing capacitated spermatozoa. Following fertilization, viable oocytes were washed twice to remove spermatozoa and cultured overnight with KSOMaa medium.

For LAIVF, one ZP perforation per fresh or V-W oocyte in FHM medium was made at room temperature on a microscope (Nikon Diaphot 200) equipped with the XYClone laser (Hamilton Thorne Biosciences, Beverly, MA) set at 100% power and 300 µs; the associated computer software was used to monitor the power and view the image. ZP perforation was performed on approximately 100 oocytes at a time and took approximately 10–15 minutes per dish. Perforated oocytes were washed twice in pre-equilibrated HTF medium and moved into an IVF drop containing spermatozoa, as described above.

### Embryo Transfer Surgery and Evaluation

The morning following IVF, all cells were counted and assessed for viability; the total number of cells, numbers of non-viable and one-cells, and number of two-cell embryos were recorded. Two-cell embryos were surgically transferred into the oviducts (14 two-cell embryos per oviduct) of the embryo transfer surgery recipients. Pseudopregnancies of recipients were induced following mating with vasectomized males 0.5 days prior to surgery; a recipient was selected based on the observation of a copulatory plug the morning of surgery.

To evaluate *in vivo* development, all recipients of embryos obtained through conventional IVF were sacrificed at embryo day 17 (E17); resorption and implantation sites were counted and the numbers of non-viable and viable E17 embryos were recorded. Approximately half of the recipients that received embryos from LAIVF were also sacrificed and evaluated as described above. The remaining embryo transfer surgery recipients were pair-housed by group and allowed to deliver pups naturally. The number of live pups delivered per recipient pair was determined, and pups were maintained for at least 2 weeks after birth.

### Statistics

Percent two-cell embryos (fertilization efficiency), viable E17 embryos, live pups and live offspring per group are presented as means ± standard error of means (SEM). Percent two-cell embryos were calculated as the number of two-cell embryos/number of total cells *100. Percent viable E17 embryos or % live pups were calculated as the number of viable E17 embryos or number of live pups/two-cell embryos transferred *100. Percent live offspring was calculated as the number of viable E17 embryos and live pups/total number of two-cell embryos transferred. All percentage data were subjected to arcsine transformation prior to statistical analysis. Data were analyzed by Student’s t-tests, where P<0.05 was considered statistically significant and 2–7 replicates were included in each group compared. Statistical analysis was performed using STATA/IC 12.1 for Mac (StataCorp, College Station, TX) and Prism Version 5.0 (GraphPad Software, La Jolla, CA).

## Results

### Vitrification of Mouse Oocytes Resulted in Satisfactory Recovery and Survival

**Figure 1:**
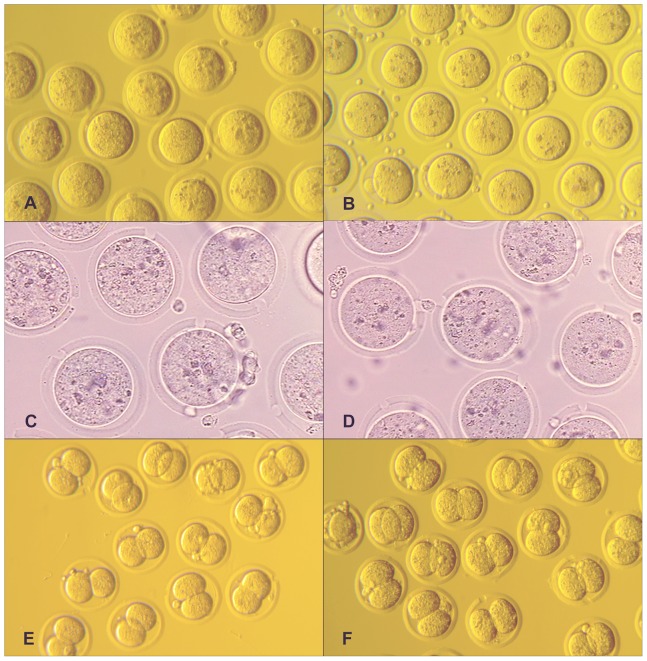
Microscopic images of V-W and fresh oocytes, and two-cell embryos generated by LAIVF. **A:** Zona-intact vitrified-warmed (V-W) oocytes, 200×. **B:** Zona-intact fresh oocytes, 200×. **C:** Zona-drilled V-W oocytes, 400×. **D:** Zona-drilled fresh oocytes, 400×. **E:** Two-cell embryos from laser-assisted IVF (LAIVF) using V-W oocytes, 200×. **F:** Two-cell embryos from LAIVF using fresh oocytes, 200×.

In total, 1,762 oocytes were vitrified, and 83% (n = 1,470) were successfully recovered from the cryogenic vials post-warming. Of those V-W oocytes recovered, 78% (n = 1,151) were viable and proceeded to be used for IVF. There were no discernible differences microscopically between survived V-W and freshly harvested oocytes before and after zona perforation, and upon generation of two-cell embryos (Figure 1).

### Cryopreservation of Mouse Spermatozoa Caused Decreased Motility, but Improved Generation of Two-cell Embryos Using Zona-drilled V-W Mouse Oocytes

The concentrations of spermatozoa in suspension obtained from each of the two donors were similar (fresh: 275×10^6^/ml; frozen-thawed, pre-freeze: 298×10^6^/ml; Table 1). Further, fresh spermatozoa from both donors had comparable motility (progressive+non-progressive motility; fresh: 74%; frozen-thawed, pre-freeze: 70%; Table 1). After freezing, the concentration of spermatozoa remained commensurate (278×10^6^/ml), but motility was decreased (35%) (Table 1).

**Table 1:** Fresh and frozen-thawed spermatozoa suspension concentration and motility from 4-month-old C57BL/6NTac proven breeders.

			Motility (%)		
Spermatozoa		aConcentration (× 10^6^/ml)	Progressive	Non-Progressive	Immotile
Fresh		275	28%	46%	26%
Frozen-Thawed	Pre-Freeze	298	47%	23%	30%
	Post-Freeze	278	21%	14%	65%

a Concentration of spermatozoa suspension per ml used for IVF was determined via a hemocytometer under light microscopy at 100× magnification, and calculated as dilution factor *count in 5 squares *0.05×10^6^, where the dilution factor was 25 [20]. Ten µl of spermatozoa suspension (approximately 3 million spermatozoa) was added to each 200 µl IVF drop of HTF medium (15 million spermatozoa/ml). At 100× magnification, the first 100 spermatozoa manually visualized were counted to establish % progressively motile, % non-progressively motile and % immotile. Based on visual observation, progressive motility was defined as spermatozoa swimming in a forward manner [21].

**Figure 2:**
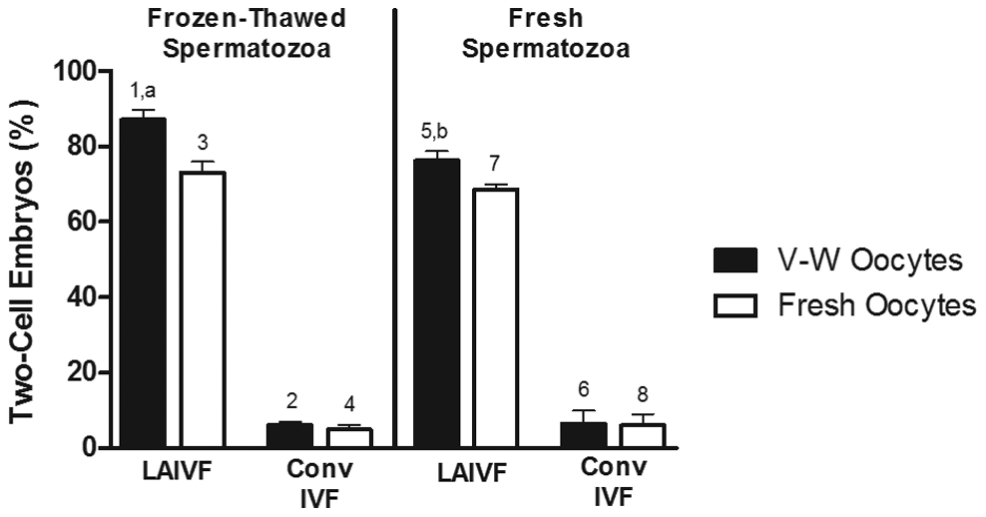
Percent two-cell embryos following LAIVF and conventional IVF using V-W and fresh oocytes. Key denotes vitrified-warmed (V-W) and fresh oocytes by black and white bars, respectively. Bars presented on the left represent data from IVF using frozen-thawed spermatozoa, and bars on the right represent data using fresh spermatozoa. Data are presented as means ± SEM. Different superscripts denote significant difference (P<0.05), where ^a-b^ denotes difference between frozen-thawed and fresh spermatozoa, and ^1–2, 3–4, 5–6, 7–8^ between laser-assisted IVF (LAIVF) and conventional IVF (Conv IVF).

Using LAIVF, V-W oocytes with frozen-thawed spermatozoa produced significantly more two-cell embryos (87.1±2.7%; Table 2) than with fresh spermatozoa (76.2±2.5%; Table 2) (P<0.05; Figure 2), but results were comparable when fresh oocytes were used. There were no significant differences in two-cell embryo efficiency between frozen-thawed and fresh spermatozoa using conventional IVF (Table 2; Figure 2).

**Table 2:** Two-cell embryo efficiency of laser-assisted IVF (LAIVF) and conventional IVF (Conv IVF) using vitrified-warmed (V-W) and fresh oocytes.

Spermatozoa	IVF	Oocytes	# Total Cells	# Non-Viable Oocytes	# One-Cells	# Two-Cell Embryos	a% Two-Cell Embryos, Mean ± SEM
Frozen-Thawed	LAIVF	V-W	343	17	28	298	87.1±2.7%
		Fresh	266	15	56	195	73.1±3.4%
	Conv IVF	V-W	168	3	155	10	5.7±1.0%
		Fresh	323	15	293	15	4.7±1.2%
Fresh	LAIVF	V-W	298	11	58	229	76.2±2.5%
		Fresh	197	9	53	135	68.5±1.8%
	Conv IVF	V-W	182	5	165	12	6.8±3.6%
		Fresh	235	4	217	14	5.7±2.9%

a Mean ± SEM of % two-cell embryos was calculated from % two-cell embryos (# two-cell embryos/# total cells *100) of each replicate; 2–4 replicates were included in each group.

### LAIVF Improved % Two-cell Embryos Using both V-W and Fresh Mouse Oocytes

In all groups compared, two-cell embryo efficiency was greater when using LAIVF than conventional IVF, with both frozen-thawed and fresh spermatozoa. For instance when frozen-thawed spermatozoa were used, LAIVF with V-W oocytes generated 87.1±2.7% two-cell embryos, while conventional IVF resulted in 5.7±1.0% two-cell embryos (P<0.001; Table 2; Figure 2). Likewise, when fresh oocytes were used, 73.1±3.4% two-cell embryos formed with LAIVF, compared to 4.7±1.2% using conventional techniques (P<0.01; Table 2; Figure 2).

### Zona-drilled V-W Mouse Oocytes Effectively Generated Two-cell Embryos

There were no statistically significant differences in using V-W versus fresh oocytes in generation of two-cell embryos. However, % two-cell embryos were higher with zona-drilled V-W oocytes compared to fresh oocytes, using both frozen-thawed (V-W oocytes: 87.1±2.7%; fresh oocytes: 73.1±3.4%; P = 0.06) and fresh spermatozoa (V-W oocytes: 76.2±2.5%; fresh oocytes: 68.5±1.8%; P = 0.12) (Table 2; Figure 2).

### LAIVF with V-W Mouse Oocytes can be Used to Produce Live Pups

**Figure 3:**
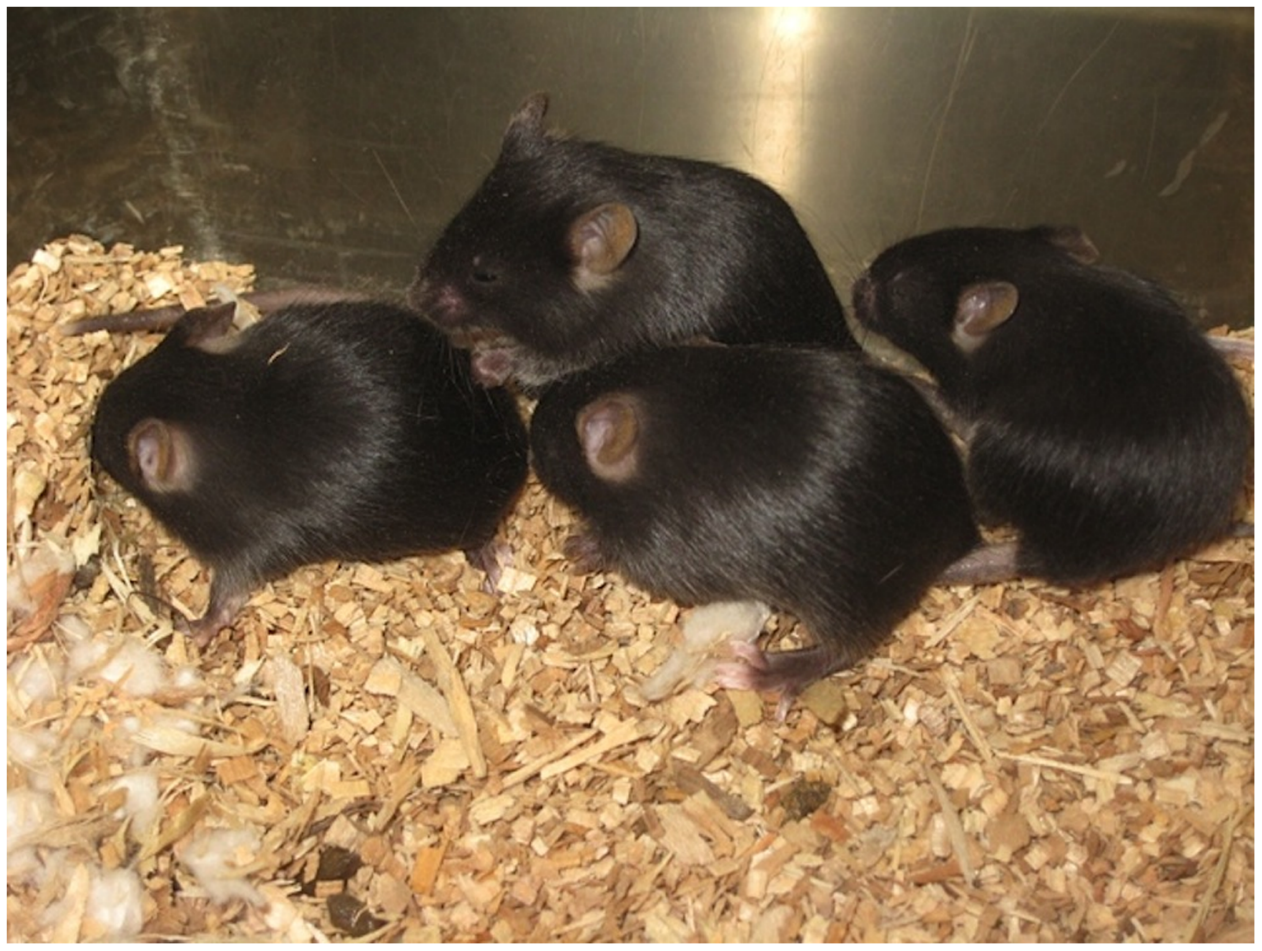
Sixteen-day-old pups from laser-assisted IVF using vitrified-warmed oocytes and fresh spermatozoa.

**Figure 4:**
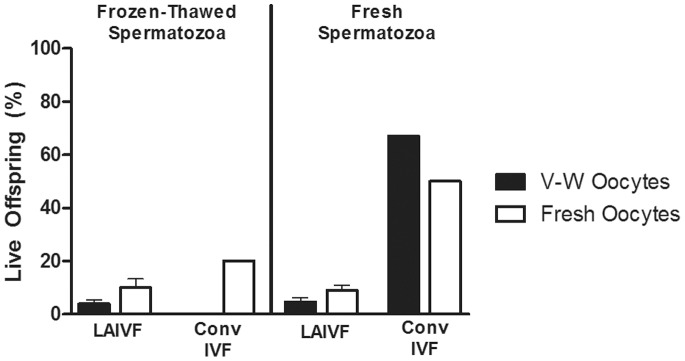
Percent live offspring following LAIVF and conventional IVF using V-W and fresh oocytes. Key denotes vitrified-warmed (V-W) and fresh oocytes by black and white bars, respectively. Bars presented on the left represent data from laser-assisted IVF (LAIVF) and conventional IVF (Conv IVF) using frozen-thawed spermatozoa, and bars on the right represent data using fresh spermatozoa. Data are presented as means ± SEM.

Normal live offspring were obtained from all groups except from conventional IVF using V-W oocytes and frozen-thawed spermatozoa, due to presumed failed induction of pseudopregnancy of the embryo transfer surgery recipient. Sixteen-day-old pups from LAIVF using V-W oocytes and fresh spermatozoa are pictured in Figure 3. While no statistically significant differences were obtained between live offspring efficiencies of zona-drilled V-W and fresh oocytes, % live offspring from fresh oocytes was higher than from V-W oocytes, using both frozen-thawed (V-W oocytes: 3.8±1.6%; fresh oocytes: 9.9±3.3%; P = 0.09) and fresh spermatozoa (V-W oocytes: 4.5±1.6%; fresh oocytes: 8.9±1.8%; P = 0.15) (Table 3; Figure 4).

**Table 3:** Live offspring efficiency of laser-assisted IVF (LAIVF) and conventional IVF (Conv IVF) using vitrified-warmed (V-W) and fresh oocytes.

			Viable E17 Embryo Efficiency						Live Pup Efficiency			
Spermatozoa	IVF	Oocytes	# Two-CellEmbryosTransf	# ResorpSites	# ImplantSites	# Non-ViableE17 Embryos	# ViableE17Embryos	a% Viable E17Embryos,Mean ± SEM	# Two-CellEmbryosTransf	b#Live Pups	a% Live Pups,Mean ± SEM	c% LiveOffspring,Mean ± SEM
Frozen-Thawed	LAIVF	V-W	112	9	1	0	2	1.8±1.8%	168	11	6.6±2.1%	3.8±1.6%
		Fresh	56	6	0	0	6	10.8±7.2%	112	10	9.0±3.6%	9.9±3.3%
	Conv IVF	V-W	10	0	0	0	0	0.0%	NA			0.0%
		Fresh	15	2	0	0	3	20.0%	NA			20.0%
Fresh	LAIVF	V-W	112	27	1	0	5	4.5±2.2%	112	5	4.5±2.7%	4.5±1.6%
		Fresh	56	8	2	0	4	7.1±0.0%	56	7	12.5%	8.9±1.8%
	Conv IVF	V-W	12	3	0	0	8	66.7%	NA			66.7%
		Fresh	14	2	0	1	7	50.0%	NA			50.0%

Resorption is abbreviated as resorp, and implantation is abbreviated as implant.

a Means ± SEM of % viable E17 embryos and % live pups were calculated from % viable E17 embryos or % live pups [# viable E17 embryos or # live pups/# two-cell embryos transferred (transf) *100] of each replicate; 2–4 replicates were included in each group where SEM is indicated.

b One replicate included number of live pups obtained from 2 embryo transfer surgery recipients co-housed together post-operatively, with a combined total of 56 transferred two-cell embryos.

c Mean ± SEM of % live offspring was calculated from % viable E17 embryos and live pups (# viable E17 embryos and live pups/total # two-cell embryos transferred *100) of each replicate; 3–7 replicates were included in each group where SEM is indicated.

NA indicates data not available; too few two-cell embryos were obtained via conventional IVF to evaluate live pup efficiency.

Statistical analysis on live offspring efficiency was not performed on groups where conventional IVF was used because too few two-cell embryos were generated to have more than one replicate per group. Overall, live offspring efficiency appeared much greater when using conventional IVF than with LAIVF: conventional IVF using V-W oocytes and fresh spermatozoa resulted in 66.7% live offspring, whereas with LAIVF, 4.5±1.6% live offspring were obtained (Table 3; Figure 4).

## Discussion

This study demonstrated that zona-drilled V-W mouse oocytes can be used for IVF procedures using both fresh and frozen-thawed spermatozoa to produce live pups. Mouse oocytes were successfully cryopreserved and recovered, and demonstrated satisfactory survival rates using DAP213 as the cryoprotectant introduced by Nakagata et al. [5], [6]. To reduce unintentional loss through adherence of V-W oocytes to the cryovial, recovery may be improved by using plastic straws or open pulled straws [8] with other cryoprotectants, although the cryovial may be preferred for temperature stability and safe transport [22]. Recent studies have investigated survival and development of cryopreserved oocytes, resulting in significant progress [1], [5], [6], [23]. For instance, one study examined a vitrification protocol of oocytes from C57BL/6J mice, and found that vitrifying COCs produced more two-cell embryos and blastocysts than cumulus-free oocytes, and vitrified COCs developed to term at a rate equivalent to the use of fresh COCs [1]. Despite the loss of oocytes to incomplete recovery and survival following vitrification, zona-drilled V-W oocytes trended toward producing more two-cell embryos than fresh oocytes. This could possibly be due to preferential selection for healthy gametes through cryopreservation; Sakamoto et al. documented that frozen-thawed oocytes were highly tolerant to damage by injection during ICSI [9]. Because two-cell embryo and live offspring efficiency are comparable between V-W and fresh oocytes, advantage can be gained using V-W oocytes in overall convenience and in reducing after-hours labor costs associated with hormone-priming and per diem rates for extended housing of oocyte donors [5].

In recent years advancements have been made in improving the efficiency of conventional IVF for both inbred and GM mice, yet imprecision and the need for technical refinement still exist. A protocol used for training in LAIVF by the European Mouse Mutant Archive reports a mean of ∼25% two-cell embryos using conventional techniques and fresh C57BL/6NTac mouse gametes [24], while another study found ∼50% to ∼80% two-cell embryos, depending on the IVF medium used [25]. In this study, we obtained 6% two-cell embryos using fresh, but cumulus-free, oocytes and spermatozoa with conventional IVF. We suspect that optimizing our technique would not only improve the percentage of two-cell embryos generated, but also show recognizable differences between using cryopreserved versus fresh gametes. One possible improvement to conventional IVF would be to maintain cumulus cell presence during culture, as this has been demonstrated to improve *in vitro* embryo development [26], [27] and fertilization efficiency [28]–[31]. One study found that the presence of an intact cumulus layer on oocytes increased the *in vitro* fertilizing ability of fresh capacitated mouse spermatozoa, with <10% of cumulus-free oocytes from GM female mice inseminated, compared to almost 40% two-cell embryos with COCs [28] Because cumulus cells must be removed for rapid and effective zona perforation, we decided to also remove cumulus cells for conventional IVF, to avoid this as a possible confounder. In an experiment performed under identical conditions, pairing instead COCs with fresh spermatozoa, we doubled the fertilization rate (14% two-cell embryos, n = 54/394; unpublished data), suggesting a disadvantage to using cumulus-free oocytes for fertilization in this study. In this same experiment, we also demonstrated improvement using fresh spermatozoa, compared to cryopreserved spermatozoa (2% two-cell embryos, n = 7/393) that are perhaps defective at penetrating the COC. Further, our use of a traditional fertilization medium (HTF) may also have contributed to lower fertilization rates obtained in this study, as HTF has been found to underperform in conventional IVF of C57BL/6 mice, compared to other media commonly used for inbred mouse strains, specifically modified Krebs-Ringer bicarbonate medium (TYH) and minimal essential medium (MEM) [25]. Other reasons the fertilization rates following conventional IVF in this study were suboptimal could be due to our selection of uniform spermatozoa samples for IVF rather than preference for the most motile, the incubator atmosphere, including a lack of nitrogen [32], and reactive oxygen species present in the fertilization milieu, perhaps from damaged frozen-thawed spermatozoa, that are known to inhibit fertilization [33]. Bath et al. found that with 4 inbred mouse strains, fertilization was increased significantly by adding reduced glutathione to the fertilization medium [33].

We found that LAIVF was significantly more efficient at producing two-cell embryos than conventional IVF using inbred mouse gametes. We recently performed a retrospective evaluation of 4 years of conventional and laser-assisted IVF data gathered from our in-house, fee-for-service operation (unpublished data). Using GM C57BL/6 mice, our fertilization success, recognizing differences in methodologies, for conventional IVF ranged from 3% to 33% two-cell embryos, depending on IVF application (mean: 12%, n = 459/3,786), generating live offspring from 7 of 45 GM lines. Whereas, LAIVF resulted in 42% two-cell embryos (n = 9,301/21,997), and was useful for obtaining live pups from 55 of 56 GM lines. Li et al. found similarly low fertilization rates using fresh spermatozoa from subfertile GM C57BL/6 mice with conventional IVF (<15% two-cell embryos), while rates using LAIVF ranged from 25% to 85% two-cell embryos [12].

As presented in Table 4, the greatest % two-cell embryos were obtained in our study with LAIVF using V-W oocytes and frozen-thawed spermatozoa (87.1%). Prior studies have evaluated various techniques to augment fertilization of V-W oocytes and demonstrated increased fertilization compared to non-treated V-W oocytes [7]–[11]. The use of oocytes zona-drilled after DAP213 vitrification in our study provided the highest % two-cell embryos reported to date using such techniques, to the best of our knowledge. While 100% of oocytes that survive ICSI with frozen-thawed spermatozoa develop to form two-cell embryos, 78% of those injected do not survive, reducing the overall % two-cell embryos to 72.3% [9]. ZP digestion using acidic Tyrode’s solution resulted in 48.8% fertilization with cryopreserved spermatozoa, compared to 22.1% without digestion [7]. Meng et al. reported a fertilization rate of 85.5% using piezo-actuated zona drilling and fresh outbred mouse spermatozoa following open pulled straw vitrification [8], while Wang et al. used zona incision to obtain 69.9% fertilization of cryopreserved oocytes with frozen-thawed spermatozoa [10]. Finally, zona drilling of oocytes prior to vitrification and subsequent pairing with fresh spermatozoa from a subfertile transgenic mouse produced a two-cell embryo rate of 52.7% [11]. Note that a recent study on a new IVF system using oocytes cryopreserved with DAP213 and specialty media alone (FERTIUP Mouse Sperm Preincubation Medium and CARD MEDIUM; KYUDO CO., LTD., Japan) reported two-cell embryo rates of 72–97% [5], showing that in prime conditions assistive techniques such as zona drilling may not be necessary to efficiently fertilize V-W oocytes.

**Table 4:** Summary of two-cell embryo and live offspring efficiencies of current and prior studies evaluating various techniques to improve penetration of the zona pellucida (ZP) of vitrified-warmed (V-W) mouse oocytes by spermatozoa.

Technique [Reference]	Spermatozoa	aDonor Mice	b% Two-Cell Embryos	c% Live Offspring
LAIVF, drilling post-vit (current study)	Frozen-Thawed	C57BL/6NTac	87.1% (298/343)	3.8% (13/280)
	Fresh		76.2% (229/298)	4.5% (10/224)
Piezo-actuated zona drilling [8]	Frozen-Thawed	Kunming	NA	NA
	Fresh		85.5% (112/131)	8.1% (12/149)
Intracytoplasmic sperm injection [9]	Frozen-Thawed	C57BL/6J Jcl	72.3% (136/188)	42.0% (68/162)
	Fresh		70.3% (78/111)	NA
Piezo-actuated zona incision [10]	Frozen-Thawed	Kunming	69.9% (137/196)	10.0% (17/170)
	Fresh		NA	NA
LAIVF, drilling pre-vit [11]	Frozen-Thawed	Oocytes: C57BL/6; Spermatozoa:C57BL/6N-Tg(UCP/FAD2)U8	NA	NA
	Fresh		52.7% (48/91)	13.3% (6/45)
Partial ZP digestion [7]	Frozen-Thawed	Kunming	48.8% (61/125)	3.4% (6/174)
	Fresh		NA	NA

Laser-assisted IVF is abbreviated as LAIVF, and vitrification is abbreviated as vit.

a Kunming mice are outbred, and C57BL/6 mice are inbred.

b Percent two-cell embryos was calculated as # two-cell embryos/# total cells post-recovery and prior to treatment *100; data presented differently in the original manuscript were reconfigured to match the format of data presentation in the current study.

c Percent live offspring was calculated as # live offspring/# two-cell embryos transferred to all recipients (pregnant and nonpregnant) *100; data presented differently in the original manuscript were reconfigured to match the format of data presentation in the current study.

NA indicates data not available.

Without a highly optimized and efficient conventional IVF, advantage was seen with using zona-drilled V-W and fresh oocytes for producing live offspring. In total, conventional IVF generated 18 live offspring from 908 oocytes (2.0%; 100% of two-cell embryos obtained were transferred), whereas LAIVF resulted in 50 live offspring using 1,010 oocytes (5.0%; 91.5% of two-cell embryos obtained were transferred). Even with optimal conventional techniques, LAIVF may still offer an advantage in circumstances where producing two-cell embryos is a challenge, such as when cryopreserved and/or GM/inbred mouse gametes are used. The % live offspring following two-cell embryo transfer documented in this study is consistent with that documented elsewhere following other treatments to improve fertilization of V-W oocytes (Table 4) [7], [8], [10], [11]. There may be a slight benefit to using zona-drilled fresh oocytes compared to V-W oocytes in producing live offspring, but this trend was not statistically significant. Meng et al. found that the rate of development to term was lower with vitrified piezo-drilled oocytes (16.6%), than with vitrified intact (36.0%) and fresh (51.3%) oocytes [8]. The advances made in this study, in terms of successful use of cryopreserved gametes with LAIVF and increased % two-cell embryos, should have an even greater impact on the production of transgenic mice once live offspring efficiency is improved.

While we weren’t able to statistically evaluate the difference between % live offspring using LAIVF and conventional IVF (and nor was this a primary focus of our study), our data suggests that more of the embryos transferred developed to produce live offspring using conventional techniques. This finding has also been demonstrated in other studies, where mouse embryos generated through laser-assisted zona drilling developed to the blastocyst stage *in vitro* at a rate similar to conventional IVF [13], [17], [18], but had significantly lower birth rates [11], [12], [17], [34], [35]. Although not ruled out here, neither parthenogenic activation, nor polyspermy appear to be problems of LAIVF with mouse gametes [11], [13], [16]–[18]. Liow et al. reported an absence of polyspermy in mouse oocytes with one laser-drilled hole in the zona paired with spermatozoa ranging in concentration from 50,000 to 2 million/ml, and only found 1% polyspermy when 2 holes were present with 1 and 2 million spermatozoa/ml [16]. While a high concentration of spermatozoa and a defect in the zona, as in our study, could both allow for more than one spermatozoon to penetrate an oocyte, zona hardening following oocyte vitrification, impaired motility of frozen-thawed spermatozoa and a precise, small hole in the zona as obtained by laser [16] could reduce the potential for polyspermy. Loss of blastomeres through the zona perforation site [10] or cytotoxic thermal damage from the laser [11], [12], [17], [34], [35] may rather be responsible for the low birth rates following LAIVF. While the true role that thermal damage plays on impaired development of embryos from LAIVF is disputed, one study found that addition of 0.25 M sucrose to the culture medium during zona drilling completely mitigated the lower birth rate [12]. Other studies have also evaluated offspring efficiency when using sucrose to osmotically shrink oocytes relative to the ZP [11], [17], [34], [35].

Frozen-thawed spermatozoa were used in this study to provide a reference for how zona-drilled V-W oocytes would perform in IVF with cryopreserved spermatozoa, with extrapolation to other spermatozoa-ZP penetration defects. Cryopreservation reduced fertilization potential of spermatozoa by decreasing the percentage of motile spermatozoa, as previously reported [6], [11], [36], [37]. The same concentration of spermatozoa was used for IVF using both fresh and frozen-thawed spermatozoa, and spermatozoa used were representative and not selective for those with good motility. The efficiency of frozen-thawed spermatozoa largely compared to that of fresh spermatozoa, but frozen-thawed spermatozoa paired with V-W oocytes for LAIVF did produce more two-cell embryos than fresh spermatozoa, suggesting again that cryopreservation may select for stronger gametes. This study demonstrates that when both male and female subfertility factors exist, LAIVF reliably ensures a high percentage of two-cell embryos and the production of healthy pups. This allows for oocyte and spermatozoa banking and shipment [5], [11], and conserves genetic resources by eliminating waste of suboptimally preserved gametes.

The ability to cryopreserve mouse gametes should considerably facilitate management of large-scale transgenic production facilities, and may ultimately support more rapid and efficient recovery of mouse lines using LAIVF. This study also contributes to the overall improvement in use of V-W oocytes in all animals, including endangered species and livestock, and in clinical applications for human assisted reproduction.
